# The Daughter—Her Health, Education and Wedlock

**Published:** 1891-05

**Authors:** 


THE DAUGHTER—HER HEALTH, EDUCATION AND WEDLOCK. Suggestions for Mothers and Daughters. By William M. Capp, M. D. Philadelphia and London. Publisher, F. A. Davis. 1891.

This little book, which is unusually attractive in appearance, and abounds in good, wholesome advice to mothers who are raising a family of children, and especially to the management of the daughters through the eventful period embraced from childhood to maidenhood. The book is intended for families, and deserves a wide circle of readers. W. A. C.

